# Social determinants of the mental health of pregnant women in Nepal: Stakeholder perspectives

**DOI:** 10.1371/journal.pone.0314736

**Published:** 2024-12-03

**Authors:** Lalita Kumari Sah, Eleni Hatzidimitriadou, Jacqueline Wier, Rajeeb Kumar Sah

**Affiliations:** 1 Faculty of Health Studies, University of Bradford, Bradford, United Kingdom; 2 Faculty of Medicine, Health and Social Care, Canterbury Christ Church University, Canterbury, United Kingdom; 3 Human and Health Sciences, University of Huddersfield, Huddersfield, United Kingdom; Birat Medical College Teaching Hospital, NEPAL

## Abstract

**Introduction:**

Women during pregnancy are at a greater risk of experiencing poor mental health, which is one of the major global public health issues, and more so in many developing countries like Nepal, but limited evidence of research on this topic is evident. In this paper, we are focused on exploring the social determinants of the mental health of pregnant women in Nepal from the stakeholder perspectives.

**Methods:**

This paper utilises eight stakeholder perspectives collected through open-ended in-depth interviews. All the interviews were analysed thematically using an inductive approach.

**Results:**

This paper presents three major findings: Inadequate social support, Limited availability and accessibility of maternal health services, and Restricted socioeconomic and cultural context. The inadequate social support from family/relatives, neighbours, community and national policies such as maternity leave, alongside the absence of NGOs/INGOs support, put women at risk of poor mental health. In addition to the inadequate support, limited availability and accessibility of maternal health services potentially cause immense distress among pregnant women. Furthermore, in the context of a patriarchal society, the impact of socioeconomic and cultural context on pregnant women’s mental health is also presented as a major determinant of poor mental health among pregnant women.

**Conclusion:**

Based on the findings, we conclude that pregnant women are at risk of poor mental health experiences in Nepal and recommend that promoting mental health among pregnant women needs a multifaceted approach that should be considered in all the policies and practices involved in promoting the mental health of pregnant women.

## Introduction

Mental health is one of the significant public health issues worldwide [1], and it is considered as an essential aspect of reproductive health [2]. Reproductive health also includes pregnant women’s experiences towards changes in their physical and mental health during pregnancy and after childbirth. The pregnancy period is a transition stage for women into motherhood when negative emotions and social changes experienced by the women can have an adverse impact on the health and well-being of the new mother and newborn babies [3]. The period from conception to two years of age timeframe is a critical stage when positive support received by mothers has a positive impact on newborn babies and provides the best possible start to their lives [4]. Several research studies have supported the argument that poor mental health during the pregnancy may cause preeclampsia in pregnancy, eclampsia in the post-natal stage, postnatal depression, decreased emotional involvement towards the child, parenting stress, premature delivery, and low birth weight of the baby [5–7]. Despite this evidence, the mental health of pregnant women has received less attention in practice and policy in the context of developing countries, and many argue that lack of research evidence, skilled workforces, and financial constraints are the main reasons [8–10]. However, there is an immense need to understand, explore and address the mental health needs of pregnant women in Nepal, which is noted and argued by experts in the subject area [11]. Hence, this paper aims to present stakeholders’ perspectives in exploring the social determinants of the mental health of pregnant women in Nepal.

In the general context of mental health, we acknowledge that several social determinants that influence mental health and well-being are responsible for increasing mental health inequalities among population groups in our society [12]. These socioeconomic circumstances, such as financial constraints, women’s positionality in society, health and social care provision, and available social support, are likely to significantly influence the mental health of pregnant women in Nepal. Deep-rooted structural issues such as gender inequalities arising from the unequal distribution of power, money, and resources further exacerbate less privileged women in a patriarchal society [13]. As in other South Asian countries [14], women in Nepal also hold the unequal distribution of caring responsibilities, which is linked to discriminatory social institutions and stereotypes on gender roles, such as looking after the family, cooking, working on their farmland, raising cattle, etc. These social circumstances could present a detrimental impact on the mental health of pregnant women as they experience limited decision-making freedom and choices, and this can adversely impact the health and well-being of the mother and newborn baby [15,16]. In this context, this paper aims to provide a broader understanding of various social determinants of the mental health of pregnant women and how these determinants such as social support, health services accessibility and availability, and socioeconomic and cultural context, can influence the mental health and wellbeing of pregnant women in Nepal from the stakeholders perspectives.

## Method

### Study design

This paper utilises findings from eight in-depth interviews with stakeholders in Nepal, which were conducted during the initial phase of the PhD research that used the mixed methods approach. [17]. The purpose of the stakeholder interviews was to gain a general understanding of the social determinants of the mental health of pregnant women in Nepal prior to conducting in-depth interviews and collecting surveys with pregnant women, which helped to explore the topic and formulate further research questions in the PhD study. In this paper, we have included open-ended, in-depth interviews with eight stakeholders.

### Data collection

The qualitative approach to collect in-depth information from a wide range of stakeholders who were experts in their subject areas provided rich data in this research using convenience and purposive sampling strategy [18]. This sampling strategy also allowed to approach stakeholders with different expertise and experiences. This research used an open-ended, in-depth interview to collect qualitative data that allowed the flexibility to explore the research questions fully until the reasons, feelings, opinions, and beliefs were fully understood during the interviews with a wide range of stakeholders [19]. All the stakeholders were encouraged to talk on the basis of the subject expertise, such as a doctor providing detailed content on health services, while a female activist spoke about socioeconomic inequalities in society and their impact on the health and well-being of pregnant women. The details of the stakeholders are provided in Table 1 below.

**Table 1:** Details of the stakeholders.

1	Nurse 1	Working in the maternity unit at district hospital
2	Nurse 2	Working as a nurse and assistant head of nursing at the hospital
3	Doctor	A Gynae/Obs doctor working at a zonal hospital
4	A nurse lecturer/An Academic	A lecturer from nursing background currently teaching nursing student
5	A previous pregnant woman	A woman with experience being pregnant and delivery baby in the past
6	A female Activist	Working for well-being and rights of women nationwide
7	Husband of current pregnant women	A man with experience of his wife during her pregnancy
8	Female Community Health Volunteer (FCHV)	FCHV is a local volunteer selected by member of health mothers’ group and their role includes advocating healthy behaviours of mothers and community people to promote safe motherhood

The qualitative interviews that include stakeholder and pregnant women took place virtually in the first phase of the study, starting from September 2020 to November 2020. All the interviews were conducted in Nepali language and translated into English by the first author (LKS), who is bilingual. Three translated qualitative interviews of the PhD research study were ‘back translated’ into the Nepali language by a bilingual researcher, to ensure the quality and accuracy of the translation [20]. The date and time for these virtual interviews were mutually agreed, and each interview with stakeholders lasted for an average of 50–60 minutes.

### Data analysis

All the interviews were transferred into NVivo 12 software, which helped to organise the data and assist in data analysis more systematically and rigorously using an inductive approach [21]. All the interviews were analysed following the six-step process of thematic analysis: Familiarising with the data; Generating initial codes; Searching for themes; Reviewing themes; Defining and naming themes; Producing tables with extract examples and themes were developed [22]. The analysis of the stakeholder interviews identified three major themes, which are presented in the results section.

### Ethical aspects

The ethics were obtained for this research from the Nepal Health Research Council (Registration No 356/2020) and Canterbury Christ Church University (Ref: ETH1920-0026). The Ilam District Hospital provided access to the fieldwork/data collection for this study. All the stakeholders provided verbal consent before starting the interviews, and the consent and interviews were recorded using a voice recorder. This research followed the guidance of both research committees in the United Kingdom and Nepal and maintained the basic principles of ethics throughout the research by following the guidelines of basic ethical principles such as respecting autonomy, beneficence, non-maleficence, and justice.

## Results

This section presents three main themes: Inadequate social support; Limited availability and accessibility of maternal health services; and Restricted socioeconomic and cultural context. On a few occasions, we have used [……..] to remove irrelevant statements so that the quotes provide focus to the themes.

### Inadequate social support

From the stakeholder’s perspective, family, relatives and neighbours’ support, organised social support, Non-Governmental Organisation and International Non-Governmental Organisation (NGO/INGO) support and maternity leave are highlighted as key factors that significantly influence the mental health of pregnant women. In the context of family and relatives support, the Obs/Gynae Doctor shared her understanding of family support and the context of pregnant women as:

*Most people, especially lower and lower-middle-class men, after marriage, the man goes back to these foreign countries for work. Men return home after 2–3 years of marriage, just for 2–3 months as a holiday. Their wives have a big pressure that they must conceive a baby by that time. Then their husband returns to work in those countries. Here, women are looked after by the husband’s family, especially in-laws. I think that is why women do not have the opportunity to share their excitement of being pregnant with their husbands and do not seem happy. These women think being pregnant is a job they should do. It looks like women are the machine to give birth to a baby. These women must have stressed that their husband is not with them, but they think it is normal as they see many women who do not have their husband with them during their pregnancy. (Obs/Gynae Doctor)*

As explained by the doctor, many women are left alone without adequate family support during their pregnancy. In many cases, limited opportunity to share emotions could mean that pregnancy is just a job without excitement, which could have a negative impact on the mental health of pregnant women and newborn babies. For those women who may not receive adequate family support, it is more stressful as no other social support is available in the community, as shared by a Nursing lecturer.

*We do recognise social issues can create huge mental distress among women, but what to do? How can we help them? We do not have a support system where nurses can refer them for further support. We recognise the problem and say, “oh poor woman” nothing more than that. We can be good listeners, that’s all. In terms of social support in the community, neighbours and community members can interfere and support if there are gender violence cases or any other visible issues. If the problem is severe, health professionals say go to the police and complain about it. In some cases, pregnant women, who do not have a family to support, are brought to the hospital by community members. […..]. As human beings, sometimes nurses collect some money and decent clothes for them and manage to refer them to a higher centre. But that is from an individual level, not from the institution level. [……….]. We do not have any organisational /institutional system to support them, nor any charities working for them. (an Academic)*

However, A Female Community Health Volunteer who was interviewed in this research suggested existence of one organised group which could be beneficial if the pregnant women are seeking financial support.

*Now AAMA SAMUHA group is active in many villages. Many groups disappear after some time, but we continue working to support each other. From the group, pregnant women get little financial support. We also have an emergency fund if women are in crisis. They can borrow some amount. We ask the young generation, daughters-in-law, to join this group, but they don’t seem interested or understand the value of this group. (A Female Community Health Volunteer)*

While exploring organisational support, none of the community/national or international-level organisations were active according to the Nursing Lecturer.

Unfortunately, I have not seen any NGOs or INGOs working to provide support to pregnant women and new mothers in this community. No organisation or group looks after or supports pregnant women in the community. I think the government should initiate such an organisation designed to support pregnant women and new mothers. (A Nursing Lecturer*)*

In addition to that, job insecurity and inadequate maternity leave could further exacerbate mental wellbeing of working pregnant women as understood by a Nursing lecturer.

*In terms of working mothers, let’s imagine, I am working in the private sector, and if I am pregnant, then I need to leave my job to spend some time with my baby and breastfeed him/her. Job insecurity is a problem for working mothers. We have a provision of paid leave for a few months, which is not sufficient, and return back to work is not guaranteed by the private sector. (A Nursing Lecturer)*

Alongside limited social support, another issue highlighted was the limited availability and accessibility of maternal health services for pregnant women, which is presented in the next subjection.

### Limited availability and accessibility of maternal health services

According to stakeholders in this research, women are likely to feel insecurity because of the limited availability and accessibility of maternal health services in Nepal. A previous pregnant woman who was interviewed in the research stated her women’s experience as:

*I was worried that because of COVID-19, the hospital may not admit me at the time of delivery. I heard doctors do not like to take the patient from other hospitals or who are not their regular patients. It was a big struggle for a pregnant woman to search for a birthing centre at that time. Women used to go here and there, looking for a place for delivery in hospitals. The first COVID death was a woman in her postnatal stage, just after the delivery of the baby, so it was a big fear of COVID among pregnant women. (Previous pregnant women)*

This issue of inadequate health facilities is further highlighted by a Female Community Health Volunteer who works in the village areas of Nepal. She also gave her statement as:

*Health posts, Gaughar Clinic (Outreach Clinic) and Khop Kendra (vaccine centre) are close to us so that women do not need to commute too far for pregnancy check-ups. However, we do not have enough health professionals in the health post for health check-ups of pregnant women. In our area, we do not have nurses who can facilitate delivery/labour. (FCHV)*

The transport system is also partly blamed by a nurse as a barrier because many women live in rural areas, and the transport system is not as convenient as women would expect. This barrier is further exacerbated when health facilities do not have adequate resources to address this challenge of geographical location and transport system. A nurse provides the context as such:

*Some women have to travel long to arrive this hospital. They say they do not have a place to stay in the town. That’s why they are not coming to the hospital very often. Women are asked to return home as soon as possible by their families. They can’t wait longer until evening. They say they can’t spend a whole day on this [………]The geographical feature creates difficulties for them (pregnant women), mainly because the transport system discourages them. (Nurse 2)*

Alongside issues of inaccessibility of the services by pregnant women, the health service also does not provide all aspects of health services. From the perspective of a Nursing lecturer, Nepal is still a highly medical model of health in practice. According to her:

*We are still at the basic level of health services. We have preventable maternal mortality issues, and we are focused on them. We are not looking at the social and emotional aspects of health. We focus on survival from ill health. Mental health is taken as an individual problem here (in the community and the hospital). We do not have a system to measure patients’ well-being in the hospital, and that is not reflected, recorded and addressed appropriately. (A Nursing Lecturer)*

The nurse working at the maternity unit gave some insight on adequate staffing in hospitals in general/ hospitals not providing adequate service as pregnant women would expect. According to the Nurse:

*Because of the workload in the hospital, sometimes some health professionals can’t explain to the women for the next health visits. The main problem, as the women say, ‘‘Nurses did not tell me about this in my last visit”. They say they did not know about it otherwise, they would come for health check-ups from the beginning of their pregnancy. Sometimes they come for the first and second health check-ups, but they do not come for the third and fourth visits. (Nurse2)*

The need to create awareness about pregnancy through antenatal education is a much-needed action in the community from the perspective of an academic who was a Nursing lecturer.

*There is a lack of adequate antenatal education. Recently, because of the safe motherhood program (Aama Surakshya Karyakram), they are aware of antenatal health check-ups, but that is not enough. For example, they may experience constipation because of the iron tablet, and abdomen bloating because of calcium tablets. To whom they should share this distress? They do not share these things with anybody, not even with health professionals as women take these distressing experiences as norms of their lives. They are not aware of hormonal and emotional changes during their pregnancy. (A Nursing Lecturer)*

Alongside awareness and resource availability in health facilities, financial constraints are another barrier which could cause immense distress to pregnant women.

*I was worried about the financial impact of delivery. I could not afford a very expensive private hospital. [………]. I looked for space in government hospitals. I could not find any space at that time, so I went to a teaching hospital. That was also so expensive for me. I thought it was a government hospital, but it was not. I could not afford a private hospital because if my baby needed NICU, which was likely, then the overall cost would be around 6 to 7 lakhs, which is too much money. There are government birthing centres, but I could not get any with the facility of the NICU at that time. (Previous pregnant women)*

There is some support available for pregnant women through the Safe Motherhood Programme, which the Nurse mentioned.

*We have an increasing number of women who have completed 4 antenatal care visits in the last 2 years, and the ‘Aama Surakshya Karyakram Programme’ is the main contributor to this uptake. In that programme, we provide incentives that include some amount of money and ‘Nyano Jhola’ (warm bag). The bag includes two sets of clothes for the newborn baby, including mittens, hats, blankets, etc., and a set of clothes for the mother. The overall cost of the bag is about NRS 1,500 (approx. £9-£10). (Nurse 2)*

However, the Female Activist challenge the Safe Motherhood Programme as an inadequate and ineffective in practice in the society where majority of the women live in. According to her:

*If a woman is pregnant in the family, then the relevant information and awareness should be given to the mother-in-law, husband, and all the members of the family. Sometimes, women cannot facilitate their desired food because of the restrictions imposed to the daughter-in-law in the family. Any awareness program should include not only pregnant women but also their family members. For example, in ‘Aama Surakyahya Karyakram’, women are entitled to get some incentives after giving birth to their baby, but we cannot guarantee that women can use the money they want. Sometimes, the mother-in-law and the husband have control over the given money. (A Female Activist)*

The context of creating awareness is further clarified by a doctor who works in maternity department in Nepal. According to her:

*Patients do not understand anything and say, and please talk to my guardian. Then we need to talk to their guardian. Women do not involve themselves in this process of decision-making. We need to focus on the family, especially in-laws, to promote the well-being of pregnant women. [….…]. We need to create awareness among the family who makes the decision on behalf of these women in the family. The family should encourage to allow women to make decisions. (Doctor)*

### Restricted socioeconomic and cultural context

This section provides an understanding of the sociocultural context in which pregnant women live and cope with circumstances during their pregnancy. From the stakeholder’s perspectives, the lowest position of the woman in the family and society, the burden of household work during pregnancy, the limited role of women in family decision-making, and the importance of giving birth to a baby boy are highly deeply rooted practices in Nepal which is shared by stakeholders in this research. There are several cultural norms that disadvantage women during their pregnancy. For example, the cultural norms that expect pregnant women to cook and feed everyone before they eat their meal can cause food insecurity in the family. The Female activist who works for women’s rights in Nepal shared her understanding as:

*A newly married woman, a daughter-in-law, eats at last when everyone finishes their eating. Our mother and our grandmother follow the same tradition. We, who live in the city, eat together. But still in our old generation, even in our generation, who live in the village areas, daughter-in-law eats at last. Sometimes, food is not left enough for women. This has been our tradition for a long time. (A Female Activist)*

This cultural norm further disadvantages women when they need to be involved in physical work without taking rest during their pregnancy. According to an academic:

*You imagine the life of a typical pregnant woman in Nepal. She works from the morning each day, working on farmland, raising cattle, cook food for the family. [….]. The women experience back pain, leg swelling, and discomfort holding the heavy weight of her baby on her tummy. Despite that, they carry a heavy weight on their back when working on farmland. (A Nursing Lecturer)*

Women activists highly criticise the practice of decision-making for women without women. Although some improvement is noted in some family circumstances, she claims the majority of women do not have decision-making freedom in the family. According to her:

*We have a patriarchal society. [……..]. Women’s decision is controlled by male members and female members of their family in some ways. Even if some women take the decision themselves, there is also patriarchal influence and practice hidden. If a husband earns money and gives that money to his wife, then the wife gets resources, and she is valued in the community. But sometimes, for that money, they experience violence as well. In the family, in-laws try to control the money, and that conflict creates gender violence. They cannot spend money on what they want. They can’t go for health-check-up when they need as their in-laws control them because their husband is not at home. It is hierarchical cultural norms. Recently women’s employment has improved a little bit, few women are in employment now, and some of those women are taking some decisions themselves. (Women activist)*

The context of decision-making and independence is further clarified by the husband of pregnant women in stakeholder interviews. According to him:

*A woman from her childhood starts behaving like a girl in terms of following her parents’ guidance, thinking about what society thinks about her, and how she can be a well-behaved girl in the family and society. In that environment, she pushes herself to be a disciplined girl, and she gets limited exposure in society, outside her home, and within her family. This societal norm restricts their experience of being independent despite their education, and the same scenario applies when they are pregnant. Pregnant women are not free from that cultural restriction. (Husband of a currently pregnant woman)*

All these cultural norms put women in a vulnerable position and at risk of violence and poor health. According to the nurse:

*Last time, a husband and wife came for treatment. It looked like the wife was beaten. Her face had scars. But the wife said her face is like this. We can’t do anything in this case until she raises her concerns. Sometimes, a woman complains that her husband married another woman because she could not give birth to a baby boy. We see the 6th, and 7th gravida because they want a baby boy. Once, I have seen 11th gravida. The reason for pregnancy was to give birth to a baby boy. If women give birth to a baby girl, then they may not be supported by the family. They say it is the same for me, boy or girl, but they celebrate differently if they have a baby boy. Still, that practice exists in our society. I don’t know what else we can do to support them. (Nurse 1)*

Overall, these quotes above present stakeholders’ perspectives that explain Nepalese pregnant women receive limited support and adequate health services and live in highly restricted cultural norms that impact their well-being.

## Discussion

From the understanding of stakeholders of this research, it is clearly visible that women in society are marginalised and experience day-to-day gender discrimination in practice and policy. Perceived social support [23] can promote the mental health of pregnant women, but it is noted that the level of support available to pregnant women may vary depending on social circumstances such as marital status, economic status, and other factors also argued in the context of different population groups [24,25]. The absence of a husband and lack of other sources of support in the community is seen as a major challenge for pregnant women, and they are at risk of experiencing poor mental health [26]. AAMA SAMUHA GROUP (Health Mother’s Group) could potentially promote the mental health of pregnant women in the community, but various challenges have been noted in the previous research [27]. There should be further exploration into whether any existing organisations are working to promote and support pregnant women in Nepal, as many stakeholders in this research claimed there is no NGO/INGO available in the community except Health Mother’s Group. In addition to that, the lack of adequate support from the national policy in terms of maternity leave further created insecurity for working women. As Nepal is progressing in promoting girls’ education, it is expected to see an increasing number of women in the service sectors, and supporting and protecting working mothers will be seen as a much-needed immediate action by the government of Nepal. Overall, from the perspective of stakeholders, pregnant women are inadequately supported and are at risk of experiencing poor mental health, which is also argued in the previous research in Nepal [28].

Accessibility and availability of adequate health services are the key concerns raised by many stakeholders in this research, which has been noted as a major issue in the majority of developing countries across the world [29]. One concern was that inadequate health professionals were present in the health facilities, which had been raised as a concern in previous research [30]. However, this is very sensitive when it comes to services related to maternal health as it is recognised that inadequate services can have a significant impact on the mother and new-born baby. Antenatal education is promoted as a key part of maternal health services in many developed countries, including the UK (National Health Services; Services), as it has a significant positive impact on maternal mental health. However, from the stakeholders’ perspectives in this research, this service is noted as less effective, and there is a lack of social and emotional support from the health professions. This certainly raises concerns regarding the standard of service Nepal is offering to pregnant women. Other concerns were focused on geographical regions where women reside and financial constraints. The majority of the population lives in rural parts of Nepal, with limited transport services to the towns and cities. Generally, well-equipped health services available in urban acres mean a large promotion of pregnant women who need well-equipped health services and are struggling to access immediate health services they need during their pregnancy, which can cause immense distress to the women. The distance, transport services, and Limited health services in the Government Hospital may push women to private health facilities, which could be unaffordable health services to the majority of Nepalese pregnant women.

From the perspective of stakeholders, it has been argued that gender discrimination in practice and policy is visible in Nepal, which is the case in many South Asian countries [31]. The woman women are seen and differentiated in practice in terms of gender preference of unborn baby by family and society, childhood, education, marriage and responsibilities to look after family after marriage have presented clear discriminatory practices in the society where pregnant women live. Limited resources and empowerment are key gender issues in Nepal as in many South Asian countries where women are restricted in decision-making of their own health and preferences [32], which significantly discourages maternal health service access [33]. Women live in social circumstances without having favourable choices, as they are dependent on their families and are considered lower in the family hierarchy. Limited empowerment and dependence also put many women at risk of violence in many South Asian countries, including Nepal [34].

## Strengths and limitations of the study

This paper presents part of a large-scale study conducted for the PhD thesis that utilised a mixed methods study design. This paper is the first of its kind to our knowledge, as none of the previous publications focused on understanding the social determinants of pregnant women in Nepal from the stakeholder’s perspectives. This paper uses findings from the eight stakeholders, which limits the generalisation of the findings and does not meet the saturation criteria given the diverse expertise of the stakeholders. However, we argue that the paper gives a snapshot of relatively unexplored areas of public mental health in the context of pregnant women from the stakeholder’s perspectives. Although the research was conducted in Nepal, we believe the knowledge shared within this paper will be referenced in many developing countries where similar socioeconomic and cultural practices are evident. Collecting the expertise and knowledge of the stakeholders was the first step to understanding the context of mental health among pregnant women, which helped the PhD research and will help many researchers to reference and explore further, mainly unexplored areas of mental health of pregnant women such as the concept of social support in pregnancy and sociocultural context. We believe the knowledge shared in this paper will play a significant role in encouraging more research to inform the development of interventions that can promote the mental health of pregnant women in Nepal and many developing countries.

## Conclusion and recommendations

In this research, we conclude that various circumstances put pregnant women at risk of poor mental health in Nepal and addressing and promoting their mental health needs a multifaceted approach, including a social approach that promotes women’s positionality and independence in society, alongside providing adequate social support and health services. The majority of women, particularly from different backgrounds, experience multiple circumstances that determine or put them at risk of vulnerability towards mental health compared to women from higher socioeconomic backgrounds. We recommend that any form of social support or organised health promotion initiative should target the most vulnerable in society. Given the context of education, income and geographical challenges, health service providers should also tailor their service to meet women’s needs. Most importantly, the policy level changes such as maternity leave and health service policy should be reviewed and implemented effectively so that women feel support from the structure and policy level. We also recommend large-scale co-production research that includes the voices of both service users and service providers before implementing any services and support for pregnant women to improve and promote their mental health.

## Supporting information

S1 File (DOCX)
